# A comparative performance evaluation of the VIDAS cytomegalovirus (CMV) interferon-gamma release assay (IGRA) and T-SPOT CMV for measuring CMV-specific T-cell responses in allogeneic hematopoietic stem cell transplant recipients receiving letermovir prophylaxis

**DOI:** 10.1128/jcm.00756-26

**Published:** 2026-07-27

**Authors:** Carlos Solano de la Asunción, Ester Colomer, Lourdes Vázquez, Irene García Cadenas, María Jesús Pascual, Pablo Granados, Carlos Martín, Olga Aso, Soukaina Boutriq, Eliseo Albert, Ariadna Pérez, José Luis Piñana, Carlos Solano, Estela Giménez, David Navarro

**Affiliations:** 1 Microbiology Service, Clinic University Hospital, INCLIVA Health Research Institute 526125, Valencia, Spain; 2 Hematology Service, Salamanca University Hospital Complex 37479, Salamanca, Spain; 3 Hematology Service, Hospital de la Santa Creu i Sant Pau 16689, Barcelona, Spain; 4 Hematology Service, Hospital Regional Universitario 16330, Málaga, Spain; 5 Hematology Service, Hospital Universitario Politécnico “La Fe” 16273 , Valencia, Spain; 6 Hematology Service, Clinic University Hospital, INCLIVA Health Research Institute 526125, Valencia, Spain; 7 Department of Medicine, School of Medicine, University of Valencia 446702, Valencia, Spain; 8 CIBER de Enfermedades Infecciosas, Instituto de Salud Carlos III 38176, Madrid, Spain; 9 Department of Microbiology School of Medicine, University of Valencia 16781, Valencia, Spain; St Jude Children's Research Hospital, Memphis, Tennessee, USA

**Keywords:** Cytomegalovirus (CMV), CMV-cell mediated immunity (CMV-CMI), VIDAS CMV interferon-gamma release assay, T-SPOT.CMV, allogeneic hematopoietic stem cell transplant recipients, letermovir prophylaxis

## Abstract

Assessment of cytomegalovirus (CMV) cell-mediated immunity (CMV-CMI) using standardized, commercially available interferon-gamma release assays (IGRAs) may improve the clinical management of CMV infection in allogeneic hematopoietic stem cell transplant recipients (allo-HCT). Studies comparing the performance of CMV IGRA assays have been conducted in this setting, revealing frequent discrepancies between assays. Here, we compared the performance of a newly released VIDAS CMV IGRA (bioMérieux) and the T-SPOT. CMV (Oxford Immunotec) was evaluated in a cohort of 85 allo-HCT recipients who received letermovir prophylaxis. A total of 107 whole blood samples were collected at either day +100 (*n* = 53) or day +180 (*n* = 54) after allo-HCT and analyzed in parallel. A moderate qualitative agreement between assays was observed (Cohen’s kappa coefficient, 0.47; 95% CI, 0.30–0.63). Discrepant results were obtained in 29 specimens, most of which were collected at day +100. A moderate correlation (rho, 0.54; *P* < 0.001) was observed between T-SPOT pp65-SPC and IFN-γ levels measured by the VIDAS CMV assay, whereas the correlation was weaker for T-SPOT IE-1 SPC and VIDAS IFN-γ levels (rho, 0.32; *P* < 0.001). The previously described booster effect of CMV DNAemia on the magnitude of CMV-CMI was captured by the VIDAS CMV assay. Neither the qualitative nor the quantitative results obtained with either IGRA predicted protection from CMV DNAemia following LMV discontinuation. While our data support the validity of the VIDAS CMV assay for the assessment of CMV-CMI in allo-HCT, they reinforce the idea that results from commercially available IGRA assays are not interchangeable, particularly in allo-HCT recipients with impaired CMV-CMI reconstitution.

**IMPORTANCE:**

This is the first study comparing a new interferon-gamma release assay (IGRA), the VIDAS cytomegalovirus (CMV), performed on whole blood and based on an enzyme-linked immunofluorescent assay detection technique, with a commercially available ELISpot assay (T-SPOT CMV) for measuring CMV-specific T-cell responses (CMV-CMI). Our cohort included allogeneic hematopoietic stem cell transplant recipients who underwent letermovir prophylaxis. In this clinical setting, tailoring the duration of LMV prophylaxis based on CMV-CMI at the time of drug discontinuation has emerged as a promising application of these assays. Our data revealed substantial qualitative and quantitative differences between the assays, which should be taken into consideration in future observational studies or clinical trials assessing the utility of IGRAs for individualizing LMV prophylaxis duration.

## INTRODUCTION

Cytomegalovirus (CMV)-specific T-cell responses (CMV-CMI) are pivotal for the prevention and control of high-level viral replication in transplant recipients, which may otherwise lead to end-organ disease (1). In this context, monitoring CMV-CMI has emerged as a useful tool for individualizing anti-CMV therapeutic strategies, either prophylactic or preemptive, in the solid organ transplantation setting (2, 3), whereas its role, if any, in allogeneic hematopoietic stem cell transplant (allo-HCT) recipients remains to be defined (4). Currently, assessment of CMV-CMI can be readily performed using commercially available interferon-gamma release assays (IGRAs) (5–7), including enzyme-linked immunospot-based tests (ELISpot) (e.g., T-SPOT CMV from Oxford Immunotec, Abingdon, UK), the QuantiFERON CMV assay (Qiagen, Hilden, Germany), and, more recently, the VIDAS CMV interferon-gamma release assay (IGRA), a fully automated ELFA (enzyme-linked fluorescent assay)-based IGRA recently launched by bioMérieux (Marcy l’Etoile, France). CMV IGRA assays are reproducible and relatively simple to perform and, with the exception of the VIDAS CMV assay, have been comprehensively evaluated in the transplantation setting proving its value in predicting the occurrence of clinically significant CMV infection (CsCMVi) (e.g., 8–19). As a result, CMV IGRA assays are prioritized over laboratory-developed flow cytometry–based assays, which often lack standardization and external validation, for assessing CMV-CMI in allo-HCT recipients (4). Studies comparing the performance of these IGRA assays in transplant recipients are scarce but consistently show that they yield qualitative and quantitative results that are not entirely interchangeable (10, 20–22). In this context, comparative studies assessing the analytical and clinical performance of CMV IGRA assays are essential for the rigorous interpretation of forthcoming clinical trials evaluating interventional strategies based on CMV-CMI measurements.

We previously compared the performance of the VIDAS CMV IGRA assay with that of a laboratory-developed, flow cytometry–based intracellular cytokine staining assay that enumerates CD4^+^ and CD8^+^ T cells targeting CMV pp65 and IE-1 and predicts the risk of CMV DNAemia following transplantation reasonably well, in a mixed cohort comprising kidney transplant and allo-HCT recipients (23). Our data supported the potential utility of the former assay for assessing CMV-CMI in transplant recipients.

The primary objective of the present study was to compare the analytical performance of the VIDAS CMV IGRA and the T-SPOT CMV assay in allo-HCT recipients receiving primary letermovir (LMV) prophylaxis, with assessment performed at the end of the scheduled prophylaxis period. In this context, immunological assessments were performed on day +100, corresponding to the standard time point for discontinuation of LMV prophylaxis (4), and on day +180, which, based on our previous experience, is the time point at which LMV is most frequently discontinued following extended LMV prophylaxis. As a secondary objective, we evaluated the clinical utility of these IGRAs for predicting late-onset CMV DNAemia, as tailoring the duration of LMV prophylaxis based on CMV-CMI at the time of drug discontinuation has emerged as a promising potential application of these assays (4, 24–32).

## MATERIALS AND METHODS

### Patients and specimens

A total of 107 whole blood samples (WB) from 85 consecutive unique adult (≥18 years old) allo-HCT recipients undergoing primary LMV prophylaxis at the five participating hospitals (Table S1) were prospectively collected between March 2025 and February 2026. Whole blood samples were collected in three lithium heparin tubes (4 mL each) at each participating site, shipped to the University Clinical Hospital of Valencia within 5 h of collection, and processed immediately upon arrival (within 18 h of collection-fresh specimens) using both CMV IGRA assays in parallel. In addition, whole blood samples from 16 healthy volunteer controls were collected from laboratory staff and processed immediately after collection. The cohort consisted of five males and 11 females, with a median age of 37 years (IQR, 28–47 years). Based on CMV IgG serology, eight individuals were CMV-seropositive, and eight were CMV-seronegative.

**Fig 1:**
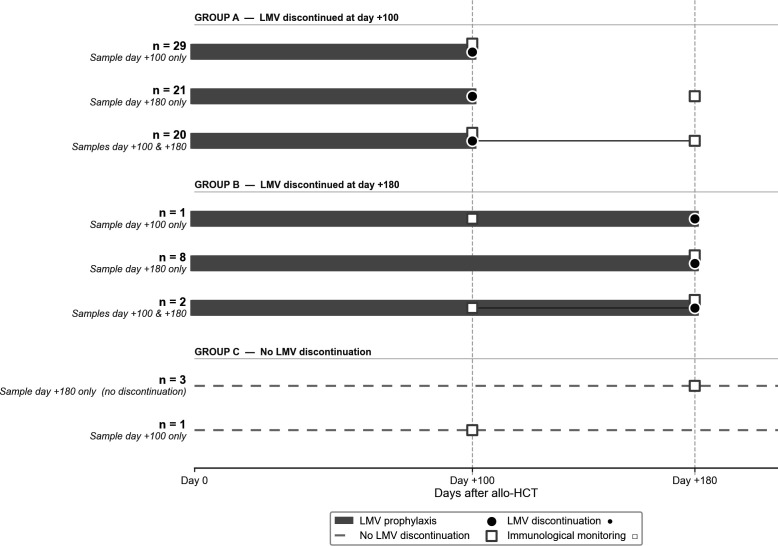
Timing of sample collection in relation to letermovir use in patients in the study cohort.

WB samples were collected on day +100 (*n* = 53) or at day +180 (*n* = 54). LMV discontinuation occurred at around +100 in 70 patients (median, 107 days; IQR, 100–125) or around +180 in 11 patients (median, 224 days; IQR, 218–307). Four patients were still under prophylaxis at the time of study completion. Twenty-two participants had WB collected at both day +100 and day +180 (Fig. 1). Criteria for the continuation of LMV prophylaxis after day +100 were aligned with the recommendations of consensus guidelines (4). Relevant information on the allo-HCT participants is displayed in Table 1. Patients were followed up for a median of 281 (IQR, 225–330) days after allo-HCT.

**TABLE 1:** Characteristics of allogeneic hematopoietic transplant patients included in the study^*b*^

Variable	No. (%)
Sex	
Female	36 (42.4)
Male	49 (57.6)
Underlying disease	
AA	3 (3.5)
ALL	7 (8.2)
AML	29 (34.1)
CLL	1 (1.2)
CML	4 (4.7)
HL	4 (4.7)
MDS/MPS	17 (20.0)
MF	7 (8.2)
NHL	12 (14.1)
Other	1 (1.2)
Prior allogeneic hematopoietic transplantation	
No	6 (5.9)
Yes	79 (92.9)
Donor type	
Related	39 (45.9)
Unrelated	46 (54.1)
Haploidentical	
No	57 (67.1)
Yes	28 (32.9)
HLA type	
Matched	35 (41.2)
Mismatched	50 (58.8)
Conditioning type	
Reduced intensity	72 (84.7)
Myeloablative	13 (15.3)
ATG use	
No	75 (88.2)
Yes	10 (11.8)
Post-transplant cyclophosphamide	
No	5 (5.9)
Yes	80 (94.1)
aGvHD prophylaxis containing sirolimus	
No	54 (63.5)
Yes	31 (36.5)
aGvHD prophylaxis containing tacrolimus	
No	34 (40.0)
Yes	51 (60.0)
aGvHD, grade	
No	46 (54.1)
Grade I	22 (25.9)
Grade II	12 (14.1)
Grade III	4 (4.7)
Grade IV	1 (1.2)
Donor CMV serostatus	
Negative	39 (46)
Positive	46 (54)
Recipient CMV serostatus	
Negative^*a*^	4 (4.8)
Positive	81 (95.2)

^*a*^ These four patients were enrolled in a clinical trial evaluating letermovir prophylaxis in CMV-seronegative patients.

^*b*^ AA, aplastic anemia; aGvHD, acute graft-versus-host disease; ALL, acute lymphocytic leukemia; AML, acute myeloid leukemia; ATG, anti-thymocyte globulin; CML, chronic myeloid leukemia; CMV, cytomegalovirus; HL, Hodgkin lymphoma; HLA, human leukocyte antigen; MDS, myelodysplastic syndrome; MF, myelofibrosis; MPS, myeloproliferative syndrome; NHL, non-Hodgkin lymphoma.

### CMV-specific immunoassays

The VIDAS CMV IGRA was performed on the VIDAS 3 system following 16-hour *in vitro* stimulation (37°C) of WB (4 mL) with a CMV lysate (VIDAS STIMM CMV RUO reagent), a mitogen (PHA-P) as a positive control (MIT), and a negative control (NIL), as previously described (23). Of relevance to the interpretation of the results described below, the viral lysates are prepared from infected cell culture supernatants without formal virus purification (personal communication from the manufacturer). The assay is calibrated against the WHO International Standard for Recombinant Human Interferon-Gamma (IFN-γ) and displays results from 0.08 to 8.00 IU/mL. All samples were processed within 30 h of collection. VIDAS CMV IGRA results were classified according to the following criteria: (i) positive samples returned positive results for both MIT and CMV lysate (≥0.2 after NIL subtraction); (ii) negative samples showed positive MIT results but negative results for both NIL and CMV lysate. Results were considered invalid when NIL values exceeded 8.0 IU/mL, while samples yielding MIT values <2.0 were classified as undetermined (Table S4). The T-SPOT CMV assay was performed using peripheral blood mononuclear cells (PBMCs) and interpreted according to the manufacturer’s instructions: (i) positive: ≥20 IE-1 and/or pp65 spot-forming cells (SPC)/250,000 PBMCs; (ii) indeterminate: <20 IE-1 and/or pp65 SPC; and (iii) negative: 0 SPC for both stimulating antigens. Spots were counted using the IRIS 1 plate reader (Mabtech AB, Nacka, Sweden) (Table S4).

### CMV serology

The CMV serological testing was performed using the DiaSorin Liaison CMV IgG assay (DiaSorin, Saluggia, Italy) according to the manufacturer’s recommendations.

### CMV plasma DNAemia quantitation and management of CMV infection

As shown in Table S1, the CMV DNA load in the plasma was quantitated using the Alinity m CMV assay (Abbott Molecular Inc., Des Plaines, IL, USA), the cobas CMV PCR (Roche Diagnostics; Indianapolis, IN, USA), the CMV ELITe MGB Kit (ELITechGroup S.p.A, Torino, Italy), or the artus CMV RG PCR (Qiagen, Hilden, Germany). Plasma CMV DNA load was monitored on a weekly basis during the first 100 days after allo-HCT; thereafter, the frequency of monitoring was progressively reduced, transitioning to biweekly and subsequently monthly intervals. Patients with CMV DNAemia at any time were monitored weekly. CMV DNA levels defining clinically significant infection (CsCMVI) ranged from 500 to 1,500 IU/mL (Table S1). CsCMVi was preemptively treated (PET) with (val)ganciclovir or foscarnet at conventional doses.

### Definitions

An episode of CMV DNAemia was defined as the detection of CMV DNA at any level (even those below the limit of quantification of the respective PCR assay-detectable/not quantifiable) in one or more plasma specimens. Episodes in which CMV DNA was detected in a single specimen, preceded and followed by a negative one, were denominated CMV DNA “blips” (33). CMV DNAemia episodes with viral DNA detected in two or more consecutive plasma specimens that cleared without PET were categorized as “self-resolving” episodes. CsCMVi encompassed episodes of CMV DNAemia that required the inception of PET, according to the center protocol, and/or CMV end-organ disease, as diagnosed according to current guidelines (34).

### Statistical analysis

Agreement between T-SPOT CMV and VIDAS CMV was assessed using positive percent agreement (PPA) and negative percent agreement (NPA), calculated as PPA = *a*/(*a* + *b*) and NPA = *d*/(*c* + *d*), where *a* represents concordant positive results, *d* represents concordant negative results, *b* represents samples positive by the comparator method but negative by the index method, and *c* represents samples negative by the comparator method but positive by the index method. Since neither method constitutes a validated reference standard, agreement was computed in both directions, with each method serving alternately as the comparator. Samples yielding indeterminate or invalid results by either assay were excluded from the agreement analysis. Exact 95% confidence intervals for PPA and NPA were calculated using the Wilson score method. Differences in positivity rates between the two methods were assessed using the McNemar test for paired proportions, with continuity correction applied. Kappa statistics were used to evaluate the degree of agreement between the comparison methods. In the healthy control cohort, a symmetric, non-directional formulation was applied: PPA = 2*a*/(2*a* + *b* + *c*) and NPA = 2*d*/(2*d* + *b* + *c*), using the same notation as above. This approach was chosen because the small and balanced sample size (*n* = 16) made the designation of one method as index or comparator arbitrary, and the symmetric formula provides a single summary estimate of agreement independent of directionality.

Differences in median values between groups were evaluated using the Kruskal-Wallis test, followed by pairwise comparisons with Bonferroni correction for multiple testing. Correlation between continuous variables was assessed using Spearman’s rank test. For analysis purposes, VIDAS IGRA IFN-γ values greater than 8.0 IU/mL were assigned a value of 10 IU/mL, while values below 0.08 IU/mL were assigned a value of 0 IU/mL. Sensitivity, specificity, negative predictive value (NPV), and positive predictive value (PPV) for predicting CMV DNAemia following LMV interruption were calculated at specific time intervals. Receiver operating characteristic (ROC) curve analysis was performed, when appropriate, to evaluate the discriminatory capacity of the test, and the area under the curve (AUC) was calculated to determine the optimal cutoff value. The cumulative incidence of CMV DNAemia and CsCMVi was estimated using the Kaplan–Meier method, with cumulative incidence calculated as 1 minus the survival function. Patients who did not experience the event of interest were censored at the time of competing events or at the last available follow-up date. Specifically, for the CsCMVi analysis, patients who developed CMV DNAemia without progressing to CsCMVi were censored at the time of CMV DNAemia detection, while patients experiencing disease relapse were censored at the time of relapse. Results are expressed as cumulative incidence percentages with 95% confidence intervals (CI). All analyses were performed using Python (version 3.13.5, with s*cipy*, *pandas*, *scikit-learn,* statsmodels, and *lifelines libraries*). Statistical significance was set at *P* < 0.05.

## RESULTS

### Performance of CMV IGRA assays in healthy individuals

WB samples from 16 healthy controls, of whom eight were CMV-seropositive and eight CMV-seronegative, were collected and tested in parallel using both CMV IGRA assays. Negative results were obtained in 8/8 and 5/8 CMV-seronegative individuals using the T-SPOT and VIDAS assays, respectively. In contrast, positive results were observed in all CMV-seropositive individuals with both assays, as well as in three CMV-seronegative subjects by the VIDAS (0.28, 7.28, and >8 IU/mL). Accordingly, PPA was 84% (95% CI, 61%–100%), NPA was 77% (95% CI, 45%–100%), and the Cohen’s kappa coefficient was 0.63 (95% CI, 0.24–1.00). IFN-γ IU/mL values generated by VIDAS correlated well and significantly with the number of IE-1 (rho, 0.70; *P* = 0.002) and pp65 (rho, 0.69; *P* = 0.003) SPC.

### Virological features of CMV DNAemia occurring during LMV prophylaxis

CMV DNAemia was detected in 40/85 (47%) patients during LMV prophylaxis at a median of 18 days (IQR, 14–44) after allo-HCT, with marked frequency differences across participating centers (Table S2). Overall, the cumulative incidence of LMV breakthrough CMV DNAemia was 47% (95% CI, 37%–59%). Most CMV DNAemia episodes (31/40) were either blips (*n* = 9) or self-resolving (*n* = 22). In 9/40 patients (22%), the CMV DNAemia episode was categorized as CsCMVi (overall cumulative incidence of 15%; 95% CI, 7.8%–27.7%). Median CMV DNA peak load in CsCMVi episodes was 3,000 IU/mL (IQR, 1,400–6,272). No CMV end-organ disease cases were diagnosed.

### Analytical performance of CMV IGRA assays in allo-HCT recipients

A total of 53 WB samples collected at day +100 and 54 collected at day +180 were assayed in parallel by both IGRA assays. Qualitative results obtained with both IGRA assays are displayed in Table 2. While the number of positive results obtained was rather comparable across both IGRA assays (*n* = 82 for T-SPOT and *n* = 77 for VIDAS; *P* = 0.09), negative results were observed more frequently with the VIDAS (*n* = 28) than with the T-SPOT (*n* = 11), although the difference did not reach statistical significance (*P* = 0.09). The difference was mainly due to both indeterminate T-SPOT results that tested negative with the VIDAS. Importantly, overall, the assays yielded discrepant results in 29/107 WB specimens (27%), including undetermined results. Detailed information about discrepancies is shown in Table S3. Most discrepancies involved positive T-SPOT results (particularly, T-SPOT pp65) with negative VIDAS results (*n* = 11) and indeterminate T-SPOT results with negative VIDAS results in specimens collected at day +100. Agreement between T-SPOT and VIDAS showed marked asymmetry depending on the direction of comparison. Using T-SPOT as the comparator, PPA and NPA were 86.4% (95% CI, 77.0–92.5%) and 72.7% (95% CI, 43.4–90.3%), respectively. When VIDAS served as the comparator, PPA was higher at 95.9% (95% CI, 88.5–98.6%), whereas NPA was substantially lower (42.1%; 95% CI, 22.1–64.7%). The Cohen’s kappa coefficient was 0.47 (95% CI, 0.30–0.63), indicating moderate concordance.

**TABLE 2:** Qualitative results obtained with the T-SPOT^*a*^^,^^*b*^

T-SPOT CMV result	VIDAS CMV result				
	Negative	Positive	Indeterminate	Invalid	Total
All samples					
Negative	8	3	0	0	11
Positive	11	70	1	0	82
Indeterminate	9	4	0	1	14
Total	28	77	1	1	107
Samples collected at day +100 after allo-HCT					
Negative	4	3	0	0	7
Positive	7	31	1	0	39
Indeterminate	5	2	0	0	7
Total	16	36	1	0	53
Samples collected at day +180 after allo-HCT					
Negative	4	0	0	0	4
Positive	4	39	0	0	43
Indeterminate	4	2	0	1	7
Total	12	41	0	0	54

^*a*^ CMV and VIDAS CMV IGRA assays in allogeneic hematopoietic stem cell transplant recipients.

^*b*^ Allo-HCT, allogeneic hematopoietic stem cell transplant; CMV, cytomegalovirus; IGRA, interferon-gamma release assay.

PPA and NPA values were higher for specimens collected at day +180 than at day +100. At day +180, PPA was 90.7% (95% CI, 78%–97%) when using T-SPOT as the reference and 100% (95% CI, 89%–100%) when using VIDAS as the reference. NPA was 100% (95% CI, 45%–100%) when using T-SPOT as the reference and 50.0% (95% CI, 19%–81.3%) when using VIDAS as the reference. At day +100, PPA was 81.6% (CI 95%: 66%–91%) when using T-SPOT as the reference and 91.2% (IC 95%: 75%–97%) when using VIDAS as the reference, and NPA was 57.1% (CI 95%: 21% – 87%) when using T-SPOT as the reference and 36.4% (CI 95%: 13%–67%) when using VIDAS as the reference. Cohen’s kappa value indicated fair agreement at both time points, with κ = 0.31 (95% CI, −0.06-0.69) at day +100 and κ = 0.41 (95% CI, 0.03–0.78) at day +180.

**Fig 2:**
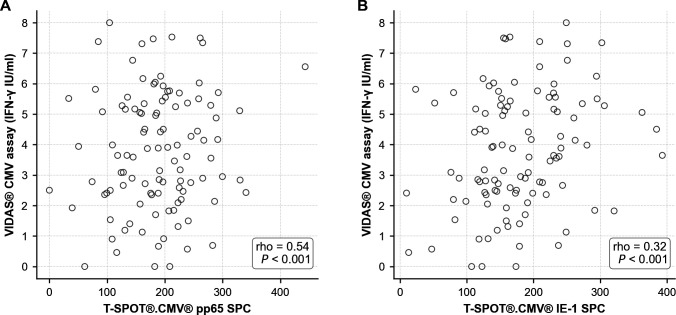
Correlation between quantitative results obtained with the T-SPOT CMV assay (in spots/250,000 peripheral blood mononuclear cells [SPC]) for pp65 (**A**) and IE-1 (**B**) and VIDAS CMV (in IFN-γ IU/mL). Rho and *P* values (Spearman rank correlation) are shown.

From a quantitative standpoint, a moderate correlation (rho, 0.54; *P* < 0.001) was observed between T-SPOT pp65-SPC and IFN-γ levels measured by the VIDAS CMV assay (Fig. 2A), whereas the correlation was weaker for T-SPOT IE-1 SPC and VIDAS IFN-γ levels (rho, 0.32; *P* < 0.001) (Fig. 2B). Interestingly, the correlation between T-SPOT IE-1 SPC values and VIDAS IFN-γ levels was higher for specimens collected at day +180 (rho, 0.45; *P* = 0.007) than for those obtained at day +100 (rho, 0.1; *P* = 0.45), whereas it was comparable at both time points for T-SPOT pp65 SPC values and VIDAS IFN-γ levels (rho, 0.56; *P* = 0.001 and rho, 0.50; *P* < 0.001, respectively).

### Impact of CMV DNAemia during letermovir prophylaxis on CMV-specific T-cell immunity

A total of 53 WB specimens from unique patients were available at day +100 for immunological testing. Prior CMV DNAemia was detected in 23 of 53 patients. Regarding measurements performed at day +180, a total of 54 WB specimens were available from unique patients, 33 of whom had developed prior CMV DNAemia. As shown in Table 3, the rate of detectable CMV-CMI at day +100 is significantly higher in patients with preceding CMV DNAemia compared to those with no documented CMV DNAemia, only when CMV-CMI was measured using the VIDAS assay (*P* = 0.04). Nevertheless, both T-SPOT IE-1 and VIDAS demonstrated a significantly higher rate of positive responses at day +180 in patients with prior CMV DNAemia compared with those in whom this event was absent (*P* = 0.04 and *P* = 0.03, respectively).

**TABLE 3:** Qualitative cytomegalovirus-specific T-cell responses measured by the T-SPOT^*c*^

IGRA assay; qualitative results (time of monitoring)	Prior CMV DNAemia^*a*^	No prior CMV DNAemia^*b*^	*P* value
Day + 100			
T-SPOT CMV result			0.68
Negative/indeterminate	3/2	4/5	
Positive	18	21	
T-SPOT CMV pp65			0.63
Negative/indeterminate	3/2	5/5	
Positive	18	20	
T-SPOT CMV IE-1			0.29
Negative/indeterminate	14/0	17/3	
Positive	9	10	
VIDAS CMV			0.04
Negative/indeterminate	3/1	13/0	
Positive	19	17	
Day +180			
T-SPOT CMV			0.28
Negative/indeterminate	1/4	3/3	
Positive	28	15	
T-SPOT CMV pp65			0.33
Negative/indeterminate	2/4	4/2	
Positive	27	15	
T-SPOT CMV IE-1			0.04
Negative/indeterminate	9/2	13/1	
Positive	22	7	
VIDAS CMV			0.03
Negative/indeterminate	4/0	8/0	
Positive	29	12	

^*a*^ *n* = 23 by day +100 and *n* = 33 by day +180.

^*b*^ *n* = 30 by day +100 and *n* = 21 by day +180.

^*c*^ CMV and the VIDAS CMV IGRA (interferon-γ release assays) at day +100 and day +180 in patients either with or without prior CMV DNAemia.

**Fig 3:**
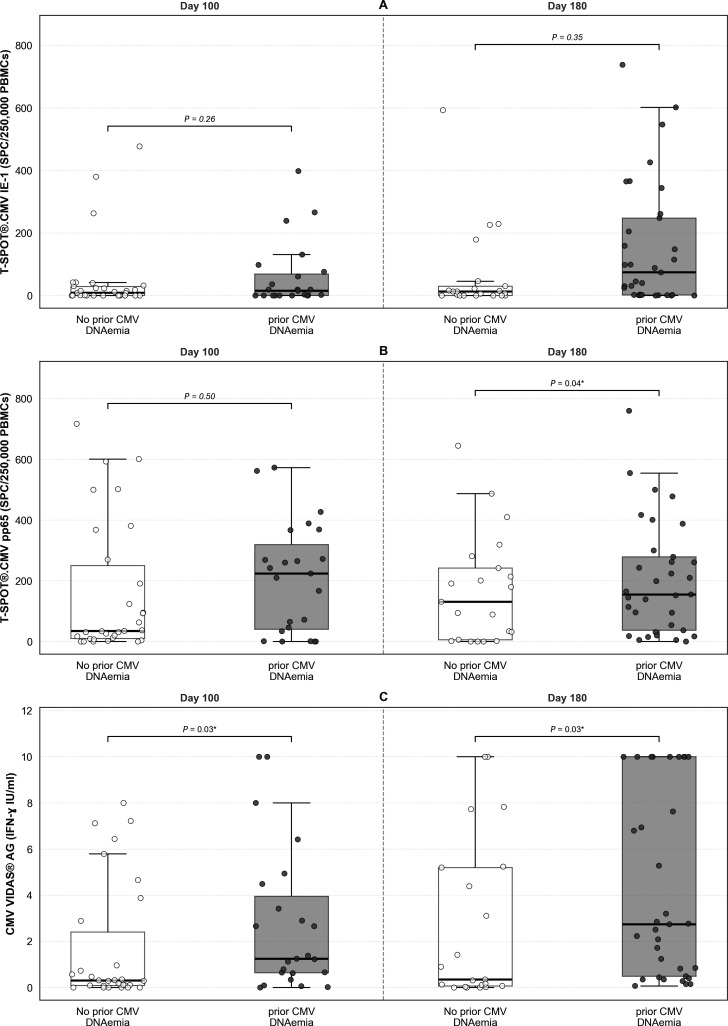
Impact of CMV DNAemia on CMV-specific cell-mediated immune responses as assessed with T-SPOT CMV pp65, T-SPOT CMV IE-1, and VIDAS CMV at days +100 and +180 after allogeneic hematopoietic stem cell transplantation in patients who underwent letermovir prophylaxis. T-SPOT CMV results are expressed as spot-forming cells (SPC)/250,000 peripheral blood mononuclear cells (PBMCs), and VIDAS CMV results are expressed in IFN-γ IU/mL. *P* values for comparisons are shown. Box plots comparing T-SPOT CMV IE-1 (A), pp65, (B) and VIDAS CMV AG (C) responses at days 100 and 180 post-transplant. Prior CMV DNAemia groups show significantly higher responses, especially at day 180.

Quantitatively (Fig. 3), median IFN-γ levels measured with the VIDAS assay were significantly higher at both time points in patients with preceding CMV DNAemia than in those without (*P* = 0.03 at both time points). In turn, prior CMV DNAemia was associated with significantly higher T-SPOT SPC only at day +180 when IE-1 SPC were considered (*P* = 0.04).

### Kinetics of CMV-specific T-cell responses as measured by both IGRA CMV assays

**Fig 4:**
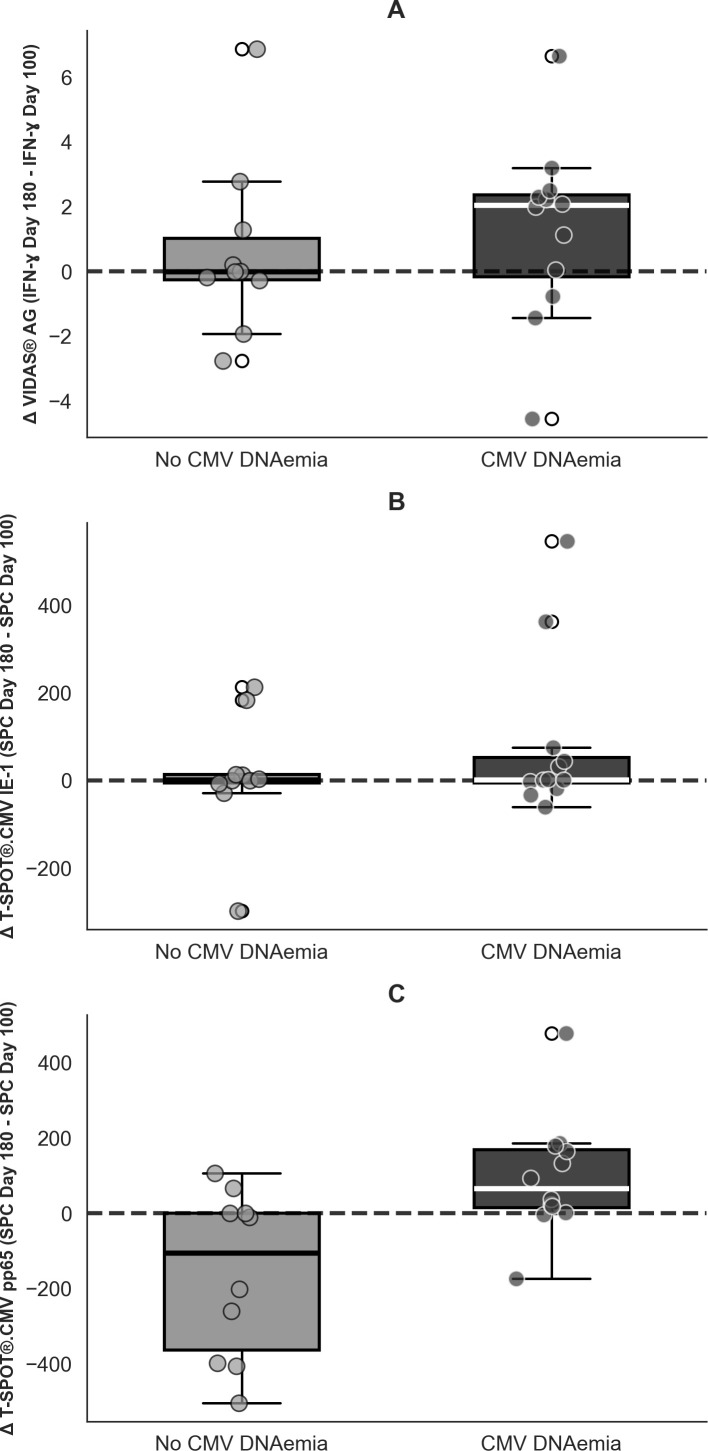
Kinetics of CMV-specific cell-mediated immune responses between days +100 and +180 after allogeneic hematopoietic stem cell transplantation, as assessed by VIDAS CMV (**A**), T-SPOT CMV IE-1 (**B**), and T-SPOT CMV pp65 (**C**). Results are expressed as the differences between testing time points (median Δ), measured in spot-forming cells (SFCs) per 250,000 peripheral blood mononuclear cells (PBMCs) for T-SPOT CMV and in IFN-γ IU/mL for VIDAS CMV.

Twenty-two patients had paired samples available at both day +100 and day +180 (Fig. 1). Notably, the proportion of patients maintaining a positive result across both time points was consistently high: 94% for T-SPOT (100% for pp65, 80% for IE-1) and 93% for VIDAS. Quantitatively, as shown in Fig. 4, patients who developed CMV DNAemia between the two time points exhibit a modest increase in VIDAS IFN-γ levels (median Δ + 2.04 IU/mL) and T-SPOT pp65 responses (median Δ + 65 SPC). In contrast, patients without CMV DNAemia showed stable or declining values across all measurements (VIDAS: Δ −0.01 IU/mL; T-SPOT pp65: Δ −105.50 SPC; T-SPOT IE-1: Δ + 1.50 SPC).

### Impact of acute graft versus host disease on GvHD on CMV-specific T-cell immunity

We next assessed whether the occurrence of severe (grades II–IV) aGvHD requiring high-dose corticosteroid therapy had any impact on CMV-specific T-cell responses, as assessed by both IGRA assays. Overall, 17 patients experienced severe aGvHD during follow-up, with the diagnosis occurring at a median of 28 days after allo-HCT (IQR, 18–47). A total of 15 and 39 WB specimens collected by day +180 from patients with severe aGvHD and without aGvHD, respectively, were available for analysis. The results are shown in Table 4. A trend toward diminished CMV-specific T-cell responses was observed in patients with severe aGvHD compared with those without aGvHD; however, statistical significance was reached only for the qualitative and quantitative T-SPOT CMV pp65 results and the quantitative T-SPOT CMV IE-1 results. Importantly, no significant differences were observed between the comparison groups in the occurrence of CMV DNAemia at any level (*P* = 0.1).

**TABLE 4:** Impact of the occurrence of severe acute graft-versus-host disease on CMV-specific T-cell responses^*a*^

Result	aGvHD (grades II–IV)		*P* value
	No (*n* = 39)	Yes (*n* = 15)	
T-SPOT CMV (qualitative)			0.08
Negative/indeterminate	5 (12%)	6 (40%)	
Positive	34 (87%)	9 (60%)	
T-SPOT CMV IE-1 (qualitative)			0.17
Negative/indeterminate	15 (38%)	10 (67%)	
Positive	24 (62%)	5 (33%)	
T-SPOT CMV pp65 (qualitative)			0.02
Negative/indeterminate	5 (13%)	7 (47%)	
Positive	34 (87%)	8 (53%)	
VIDAS CMV result (qualitative)			0.22
Negative	8 (21%)	5 (33%)	
Positive	31 (79%)	10 (67%)	
T-SPOT CMV IE-1, median SPC (IQR)	40 (6.5–228)	2 (0–57)	0.01
T-SPOT CMV pp65, median SPC (IQR)	199 (92–354)	20 (5.5–131)	0.005
VIDAS CMV, median IFN-γ in IU/mL (IQR)	2.23 (0.35–7.68)	1.24 (0.21–6.11)	0.63

^*a*^ IU, international units; SPC, spot-forming cells/250,000 PBMCs.

### CMV IGRA assays for predicting CMV DNAemia after letermovir prophylaxis discontinuation

Overall, CMV DNAemia developed in 25 patients after LMV interruption at a median of 149 days after allo-HCT (IQR, 135–178). Of these, 18 cases occurred between day +100 and day +180, and seven cases occurred thereafter. The median peak CMV DNA load was 376 IU/mL (IQR, 143–1,034 IU/mL). In six patients (27%), CMV DNAemia was categorized as CsCMVi (three occurring before and three after day +180).

**Fig 5:**
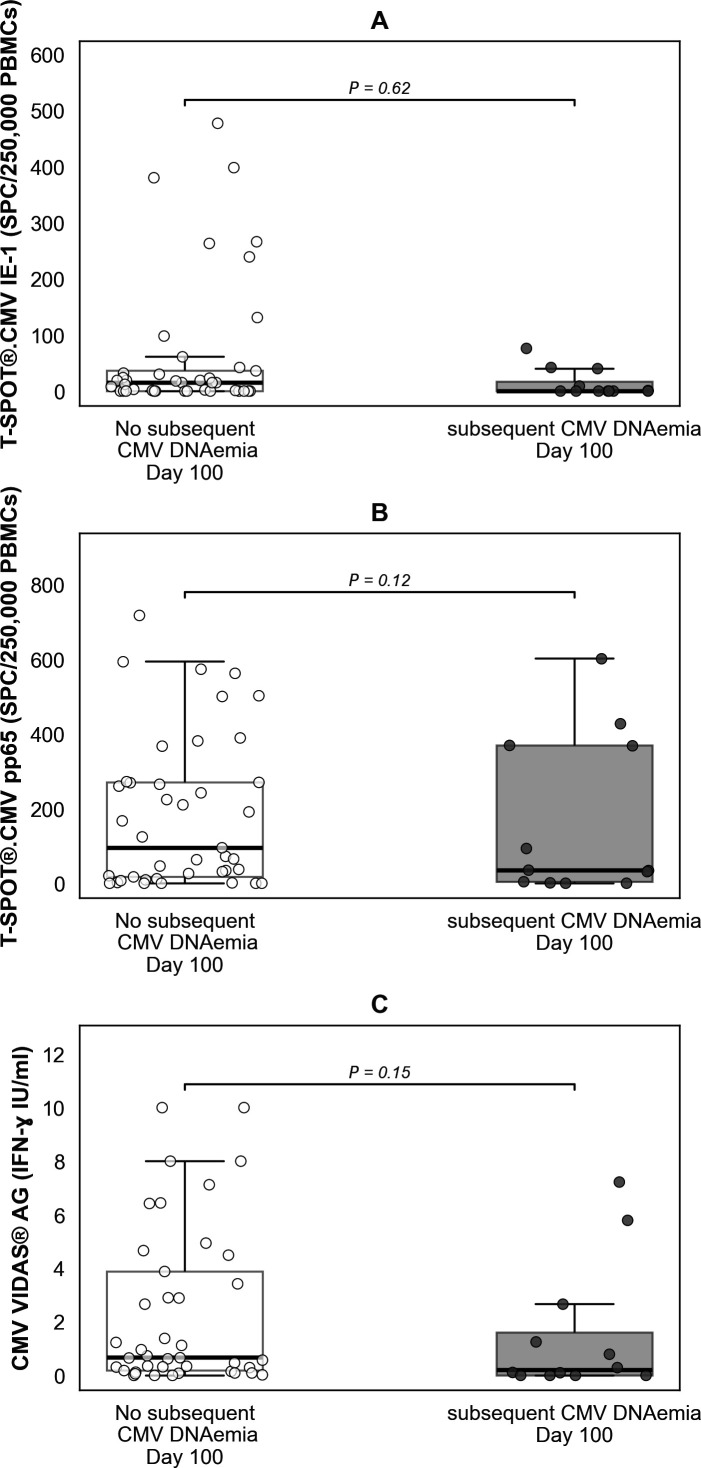
CMV-specific cell-mediated immune responses at the end of letermovir prophylaxis (around day +100), as measured by T-SPOT CMV IE-1 (**A**), T-SPOT CMV pp65 (**B**), and VIDAS CMV (**C**), and their association with subsequent CMV DNAemia. *P* values for comparisons are shown.

Qualitatively, regardless of the IGRA CMV assay employed, there was no difference in the rate of positive or negative results at day +100 between patients who did or did not subsequently develop CMV DNAemia (Table 5; Fig. 5). Positive IGRA results—whether measured by T-SPOT or VIDAS—showed high positive predictive values for protection against post-LMV CMV DNAemia (PPV range, 79–84%). Conversely, negative predictive values were low (NPV range, 24–35%), suggesting that a negative test result does not reliably identify patients at risk of CMV DNAemia. When analyzing CMV-specific T-cell responses quantitatively, no significant differences were observed between patients who developed post-LMV CMV DNAemia and those who did not. Median T-SPOT IE-1 SPC, pp65 SPC, and VIDAS IFN-γ values were numerically higher in the non-CMV DNAemia group, but differences did not reach statistical significance (*P* = 0.62, *P* = 0.12, and *P* = 0.15, respectively) (Fig. 5).

**TABLE 5:** Qualitative cytomegalovirus-specific T-cell responses measured by the T-SPOT^*a*^^,^^*b*^

IGRA assay (time of monitoring)	Post-LMV CMV DNAemia	No post-LMV CMV DNAemia	*P* value	PPV (95% CI)	NPV (95% CI)
T-SPOT CMV (+100), no. positive/no. negative	8 (67%)/4 (33%)	31 (75%)/10 (24%)	0.81	79.5% (63.5–90.7)	28.6% (8.4–58.1)
T-SPOT CMV IE-1 (+100), no. positive/no. negative	3 (25%)/8 (75%)	16 (39%)/25 (61%)	0.58	84.2% (60.4–96.6	24.2% (11.1–42.3)
T-SPOT CMV pp65 (+100), no. positive/no. negative	8 (67%)/4 (33%)	30 (73%)/11 (27%)	0.94	78.9% (62.7–90.4)	26.7% (7.8–55.1)
VIDAS CMV (+100), no positive/no. negative	6 (50%)/6 (50%)	30 (73%)/11 (27%)	0.25	83.3% (67.2–93.6)	35.3% (14.2–61.7)

^*a*^ CMV and the VIDAS CMV IGRA (interferon-γ release assays) at time points +100 and +180, and subsequent occurrence of CMV DNAemia.

^*b*^ Indeterminate and invalid results are computed as negatives. CMV, cytomegalovirus; LMV, letermovir.

## DISCUSSION

There is increasing evidence suggesting that the magnitude of CMV-CMI measured in allo-HCT recipients at the time of LMV prophylaxis interruption may categorize patients as being at high or low risk for late-onset CsCMVi. Accordingly, assessment of CMV-CMI may help determine whether LMV prophylaxis should be prolonged or discontinued as planned (4). The aforementioned evidence has been mainly obtained using laboratory-developed, flow cytometry–based intracellular cytokine staining (FC-ICS) assays (24–27), which lack rigorous standardization and external validation and produce data that are difficult to compare. The use of commercially available CMV IGRA tests is preferred over FC-ICS assays for generating evidence on the potential utility of CMV-CMI assessment in the management of CMV infection in the allo-HCT setting, owing to their higher degree of standardization (4). Nevertheless, the analytical qualitative and quantitative results returned by CMV IGRA assays are often inconsistent (10, 20–22). Moreover, the clinical value of CMV IGRA assays in predicting the occurrence of CsCMVi following LMV discontinuation has also been shown to vary, with ELISpot assays performing substantially better (25, 29, 30) than the QuantiFERON-CMV assay (35). Recently, the VIDAS CMV assay has been introduced to the market; it offers several operational advantages over ELISpot assays, including minimal hands-on time (approximately 5 min), automation, operational simplicity, and a whole-blood workflow that obviates the need for PBMC isolation. Data from our group supported its potential utility for assessing CMV-CMI in transplant recipients (23). The main purpose of the current study was to compare, from an analytical standpoint and for the first time, the VIDAS CMV assay and the T-SPOT CMV test in a cohort of CMV-seropositive (mainly) allo-HCT recipients undergoing primary LMV prophylaxis. Our data clearly showed that the qualitative and quantitative results provided by the two assays are not interchangeable. This finding is consistent with previous studies comparing the performance of IGRA assays across different clinical settings (10, 20–22). In detail, discrepant qualitative results between the T-SPOT and VIDAS assays were observed in approximately one third of the specimens, most of which consisted of positive T-SPOT pp65 results with negative VIDAS results or indeterminate T-SPOT results with negative VIDAS results. This resulted in a Cohen’s kappa coefficient of 0.47, indicating moderate concordance.

These data point to a higher sensitivity of the T-SPOT CMV assay, which may appear counterintuitive at first glance. Indeed, whereas T-SPOT CMV uses immunogenic peptides derived from the non-structural viral protein IE-1 and the structural protein pp65, the VIDAS CMV assay employs a whole-virus lysate containing pp65 and other structural components, such as envelope glycoproteins, but notably lacking IE-1 because of its non-structural nature (although the presence of trace amounts of IE-1 in viral lysates capable of stimulating T cells cannot be completely excluded). This difference in antigen composition may, at least in part, contribute to the observed discordance between the assays.

IE-1 is one of the most immunodominant targets of CMV-specific T-cell responses, particularly for functional CD8^+^ T cells (1). Therefore, its inclusion in the T-SPOT CMV panel, but most likely not in the VIDAS CMV lysate, could explain why T-SPOT CMV detects CMV-specific responses in a subset of patients that the VIDAS assay fails to identify. Conversely, the broader antigenic repertoire of the viral lysate, encompassing multiple structural proteins, may stimulate responses that are not captured by the peptide-based T-SPOT CMV panel, potentially accounting for cases in which the VIDAS assay yields positive results while T-SPOT CMV does not. Furthermore, procedural differences between the assays—particularly the use of isolated PBMCs in the T-SPOT assay versus whole blood in the VIDAS assay, as well as differences in antigen stimulation periods—may, at least in part, account for the above observation. Likewise, cocktails of highly immunogenic peptides may elicit stronger combined CD4^+^ and CD8^+^ T-cell responses compared to virus lysates, which predominantly stimulate CD4^+^ T cells, as soluble antigens are more efficiently presented through the exogenous MHC class II pathway (36, 37). Taken together, these antigenic and procedural differences likely account for at least part of the substantial variation observed between the T-SPOT CMV and VIDAS CMV IGRA results in our cohort.

In support of this view, the highest concordance between a laboratory-developed ELISpot assay and the VIDAS assay in a recent study (38) was observed with CMV pp65 + IE-1 peptide pools (100%), followed by the pp65 peptide pool (91.7%) and the virus lysate (80%). Nevertheless, in our study, agreement between the T-SPOT and VIDAS assays showed marked asymmetry depending on the direction of comparison, reflecting the imbalance in the proportions of positive and negative results yielded by each method. The wide confidence intervals for NPA estimates, particularly in the VIDAS-referenced analysis, reflect the limited number of concordant negative pairs (*n* = 8) and should therefore be interpreted with caution.

Interestingly, PPA and NPA values were higher for specimens collected at day +180 than for those collected on day +100, with the kappa value also being higher at the former time point (κ = 0.41 vs κ = 0.31, respectively). Although speculative, this could be related to the higher level of CMV-specific T-cell reconstitution that occurs at later time points following allo-HCT.

Quantitatively, the correlation between T-SPOT SPC and VIDAS IFN-γ levels was moderate for T-SPOT pp65-SPC (rho, 0.54) and weaker for T-SPOT IE-1 (rho, 0.32). Although we do not provide experimental evidence to support this hypothesis, one possible explanation is that the VIDAS viral lysate contains substantial amounts of pp65 and, at most, trace amounts of IE-1. Consequently, IE-1-specific T-cell responses detected by the T-SPOT CMV assay would not be expected to contribute substantially to the IFN-γ signal measured by the VIDAS CMV assay. In addition, IE-1 preferentially elicits CD8^+^ T-cell responses, whereas pp65 stimulates both CD8^+^ and CD4^+^ T cells (1). This, together with the fact that, as discussed above, the T-SPOT CMV assay captures both CD8^+^ and CD4^+^ T-cell responses, while the VIDAS CMV assay predominantly reflects CD4^+^ T-cell activity (36, 37), may account for our observations.

A stronger correlation was observed in specimens collected on day +180 than in those obtained on day +100. Taken together, these data suggest that concordance and correlation between the two assays are likely more consistent in patients with a higher degree of CMV-CMI reconstitution (day +180). Of relevance, despite differences in both qualitative and quantitative results between the two assays, the kinetics of CMV-CMI assessed in 22 paired whole-blood specimens obtained at days +100 and +180 appeared rather similar, particularly for T-SPOT pp65 SPC.

An observation that warrants comment is that, while all eight CMV-seronegative healthy individuals showed negative responses by the T-SPOT CMV assay, three tested positive with the VIDAS CMV assay. A similar finding has been previously reported using both the VIDAS CMV assay and an in-house flow cytometry assay enumerating CD4^+^ and CD8^+^ T cells specific for pp65 and IE1 (23), as well as using the VIDAS CMV assay employing pp65 peptides as the stimulating antigen (38). Although these results may represent false-positive VIDAS CMV results, an alternative explanation is that they reflect false-negative serological results in individuals previously infected with CMV who failed to maintain detectable CMV IgG responses. Such discordances between CMV serology and CMV-specific T-cell immunity have been described in both immunocompetent and immunosuppressed individuals and may primarily be explained by differences in the analytical sensitivity of serological and T-cell assays. Nevertheless, the small sample size of our study precludes definitive conclusions regarding this observation, which warrants further investigation in adequately powered studies.

The use of high-dose corticosteroids for the treatment of severe aGvHD in patients receiving LMV has been associated with impaired CMV-specific T-cell reconstitution, even after adjustment for the occurrence of breakthrough CsCMVi (24, 30). Here, we assessed whether this effect could be captured by the IGRA assays under comparison. Our data indicated that, as expected and irrespective of the occurrence of CMV DNAemia, patients who developed severe aGvHD exhibited lower CMV-specific T-cell responses at day +180. However, statistically significant differences were observed only for selected T-SPOT CMV results. These findings should be interpreted with caution given the limited number of patients included in the analysis. This limitation also precluded the corresponding analysis at day +100.

LMV is known to impair the reconstitution of CMV-CMI in allo-HCT recipients (24–27). In this context, it has been shown that the occurrence of breakthrough CMV DNAemia, particularly CsCMVi, boosts CMV-specific immune responses as assessed by both FC-ICS and T-SPOT assays (24–27, 29, 39). We next investigated whether the VIDAS CMV assay could capture this effect. Indeed, the rate of detectable CMV-CMI at both day +100 and day +180 was significantly higher in patients with preceding CMV DNAemia compared with those without CMV DNAemia. Furthermore, median IFN-γ levels measured with the VIDAS assay were significantly higher at both time points in patients with preceding CMV DNAemia than in those without it. In contrast, the impact of CMV DNAemia on the rate of detectable responses or their magnitude in the T-SPOT assay was only consistent for T-SPOT IE-1. Likewise, the occurrence of CMV DNAemia boosted IFN-γ levels measured with the VIDAS assay at both time points; however, when using the T-SPOT assay, this effect was only observed at day +180 for IE-1 SPC. Finally, the ability of both IGRA assays to predict the occurrence of late-onset (post-LMV) CMV DNAemia was investigated. Previous studies using the T-SPOT CMV assay reported significantly higher levels of either pp65 SPCs (29) or both pp65 and IE-1 SPCs (30) in patients who were protected from late-onset CsCMVi. In our cohort, we failed to reproduce those results for CMV DNAemia; however, a quantitative trend toward stronger responses was observed with the T-SPOT pp65 SPC and VIDAS assays. In contrast to the aforementioned studies (29, 30), we included all CMV DNAemia episodes, whether self-resolving or CsCMVi; the small number of the latter in our cohort precluded separate analysis. Additionally, differences in patients’ clinical characteristics, the variable duration of LMV prophylaxis, the timing of immunological testing, the PCR employed, and CMV DNA thresholds for CsCMVi categorization may account for the discrepant results. Major limitation of the present study is the relatively small sample size, particularly in subgroup analyses stratified by time point and DNAemia status, which precluded robust subanalyses, such as assessing the impact of severe aGvHD or according to the type of allo-HCT. Likewise, we acknowledge that the healthy control group was not formally powered and that the limited number of individuals represents a constraint of this study. The inclusion of healthy controls was intended to provide a reference framework for assay performance rather than a formal diagnostic accuracy analysis.

### Conclusion

Our data support the validity of the VIDAS CMV assay for measuring CMV-CMI in allo-HCT and further reinforce the idea that commercially available CMV IGRA assays may yield disparate qualitative and quantitative results. To definitively elucidate the clinical utility of these assays, further multicenter, side-by-side studies in homogeneous cohorts with defined time points for immunological testing are warranted.

## Data Availability

The data sets generated and/or analyzed in the current study are available from the corresponding author upon reasonable request.

## SUPPLEMENTAL MATERIAL

Supplemental tables jcm.00756-26-s0001.docx Tables S1 to S4.

ASM does not own the copyrights to Supplemental Material that may be linked to, or accessed through, an article. The authors have granted ASM a non-exclusive, world-wide license to publish the Supplemental Material files. Please contact the corresponding author directly for reuse.
