# Supplementary material for: A comparative performance evaluation of the VIDAS cytomegalovirus (CMV) interferon-gamma release assay (IGRA) and T-SPOT CMV for measuring CMV-specific T-cell responses in allogeneic hematopoietic stem cell transplant recipients receiving letermovir prophylaxis
Source: J Clin Microbiol. 2026 Jul 27;64(8):e00756-26. doi: 10.1128/jcm.00756-26 (PMC13463899; doi:10.1128/jcm.00756-26)
Supplement: Supplementary file 1 [file jcm.00756-26-s0001.docx]

| **Supplementary Table 1. Hospital participants,number of patients included and PCR used for CMV surveillance.** | | | | |
| --- | --- | --- | --- | --- |
| **Center** | **Number of patients included** | **PCR used for CMV surveillance** | **LOD (IU/ml)** | **Threshold for PET (IU7ml)** |
| *Hospital de la Santa Creu i Sant Pau, Barcelona* | 28 (33%) | CMV ELITe MGB® Kit (ELITechGroup) | 88 – 17 (depending on input sample volume) | >1000 (two consecutive) |
| *University Clinical Hospital of Valencia, Valencia.* | 22 (26%) | Alinity m CMV assay  (Abbott Molecular) | 30 | 1500 |
| *Salamanca University Hospital Complex, Salamanca* | 18 (21%) | cobas® CMV PCR  (Roche Diagnostics) | 34.5 | 1000 |
| *Regional University Hospital of Malaga, Malaga* | 12 (14%) | Alinity m CMV assay  (Abbott Molecular) | 30 | 500 |
| *The University and Polytechnic Hospital of L*a Fe | 5 (5.9%) | Artus CMV RG PCR KIt | 145 | 500 |

| **Supplementary Table 2. Occurrence of CMV DNAemia Under LMV Prophylaxis and Following LMV Discontinuation in Participating Centers.** | | | | |
| --- | --- | --- | --- | --- |
| **Center** | **CMV DNaemia under LMV (n=85)** | **CsCMVi under LMV (n=85)** | **CMV DNAemia after LMV discontinuation (n=25)** | **CsCMVi after LMV (n=6)** |
| *Hospital de la Santa Creu i Sant Pau, Barcelona (n=28)* | 10 (36%) | 5/10 (50%) | 6 (21%) | 1/6 (17%) |
| *University Clinical Hospital of Valencia, Valencia (n=22)* | 18 (82%) | 2/18 (11%) | 7 (32%) | 1/7 (14%) |
| *Salamanca University Hospital Complex, Salamanca (n=18)* | 6 (33%) | 1/6 (17%) | 5 (28%) | 1/5 (20%) |
| *Regional University Hospital of Malaga, Malaga (n=12)* | 2 (17%) | 0/2 | 5 (42%) | 2/5 (40%) |
| The University and Polytechnic Hospital of La Fe (n=5) | 4 (80%) | ¼ (25%) | 2 (40%) | ½ (50%) |

| **Supplementary Table 3. Discordant results between T-SPOT® and CMV VIDAS® IGRA results** | | | | | |
| --- | --- | --- | --- | --- | --- |
| **T-SPOT result** | **VIDAS result** | **Positive recipient’s CMV serostatus** | **T-SPOT IE** | **T-SPOT pp65** | **VIDAS Ag** |
| Negative | Positive | Yes | 0 | 0 | 0.29 |
| Negative | Positive | Yes | 0 | 0 | 4.49 |
| Negative | Positive | Yes | 0 | 0 | 0.66 |
| Positive | Negative | Yes | 380 | 593 | 0.15 |
| Positive | Negative | Yes | 40 | 35 | 0.10 |
| Positive | Negative | Yes | 115 | 31 | 0.15 |
| Positive | Negative | Yes | 0 | 34 | 0.02 |
| Positive | Negative | Yes | 23 | 12 | 0.08 |
| Positive | Negative | Yes | 30 | 31 | 0.12 |
| Positive | Negative | Yes | 6 | 32 | 0.17 |
| Positive | Negative | Yes | 229 | 214 | 0.00 |
| Positive | Negative | Yes | 42 | 34 | 0.00 |
| Positive | Negative | Yes | 0 | 31 | 0.00 |
| Positive | Negative | Yes | 36 | 260 | 0.09 |
| Positive | Undetermined | Yes | 61 | 167 | 0.02 |
| Undetermined | Positive | No | 3 | 17 | 0.44 |
| Undetermined | Negative | Yes | 15 | 9 | 0.18 |
| Undetermined | Negative | Yes | 0 | 2 | 0.00 |
| Undetermined | Negative | Yes | 1 | 18 | 0.15 |
| Undetermined | Negative | Yes | 16 | 2 | 0.07 |
| Undetermined | Negative | Yes | 1 | 7 | 0.09 |
| Undetermined | Negative | No | 0 | 2 | 0.13 |
| Undetermined | Negative | Yes | 2 | 5 | 0.07 |
| Undetermined | invalid | Yes | 0 | 6 | 0.03 |
| Undetermined | Positive | Yes | 18 | 17 | 0.35 |
| Undetermined | Negative | Yes | 0 | 4 | 0.00 |
| Undetermined | Positive | Yes | 2 | 5 | 2.09 |
| Undetermined | Positive | Yes | 0 | 1 | 2.66 |
| Undetermined | Negative | Yes | 0 | 1 | 0.06 |

| **Supplementary Table 4. Interpretation criteria for VIDAS® CMV IGRA and T-SPOT®.CMV assays** | | | |
| --- | --- | --- | --- |
| **VIDAS® CMV IGRA** | | | |
| **Result** | **NIL (negative control)** | **MIT (mitogen control)** | **CMV lysate (after NIL subtraction)** |
| **Positive** | ≤8.0 IU/mL | ≥2.0 IU/mL | ≥0.2 IU/mL |
| **Negative** | ≤8.0 IU/mL | ≥2.0 IU/mL | <0.2 IU/mL |
| **Invalid** | >8.0 IU/mL | — | — |
| **Indetermined** | ≤8.0 IU/mL | <2.0 IU/mL | — |
| **T-SPOT®.CMV** | | | |
| **Result** | **IE-1 SPC / 250,000 PBMCs** | **pp65 SPC / 250,000 PBMCs** | **Decision rule** |
| **Positive** | ≥20 SPC | Any value (≥0 SPC) | IE-1 ≥20, regardless of pp65 |
| **Positive** | Any value (≥0 SPC) | ≥20 SPC | pp65 ≥20, regardless of IE-1 |
| **Indeterminate** | >0 but <20 SPC | 0 SPC or >0 but <20 SPC | IE-1 between 1–19; pp65 not ≥20 |
| **Indeterminate** | 0 SPC or >0 but <20 SPC | >0 but <20 SPC | pp65 between 1–19; IE-1 not ≥20 |
| **Negative** | 0 SPC | 0 SPC | Both IE-1 and pp65 = 0 |
| SPC: spot-forming cells; NIL: negative control; MIT: mitogen positive control; PBMCs: peripheral blood mononuclear cells; IU: international units*.* | | | |
